# The key role of Shenyan Kangfu tablets, a Chinese patent medicine for diabetic nephropathy: study protocol for a randomized, double-blind and placebo-controlled clinical trial

**DOI:** 10.1186/1745-6215-14-165

**Published:** 2013-06-05

**Authors:** Hui Wang, Wei Mu, Jingbo Zhai, Dongmei Xing, Shujie Miao, Jia Wang, Yueyi Deng, Niansong Wang, Hongyu Chen, Hongtao Yang, Xuehong He, Hongcai Shang

**Affiliations:** 1 Tianjin Institute of Clinical Evaluation, Tianjin University of TCM, 88 Yuquan Street, Nankai District, Tianjin 300193, China; 2 Tianjin Tongrentang Group Co, Ltd., Machang Street, Hexi District, Tianjin 300132, China; 3 Department of Nephrology, Longhua Hospital of Shanghai University of TCM, 725 South Wanping Road, Shanghai 200032, China; 4 Department of Nephrology and Rheumatology, The Sixth People's Hospital of Shanghai, 600 Yishan Road, Shanghai 200233, China; 5 Department of Nephrology, Guangxing Hospital Affiliated to Zhejiang University of TCM, Tiyuchang Road, Hangzhou 310007, China; 6 Department of Nephrology, The First Affiliated Hospital of Tianjin University of TCM, 314 West Anshan Road, Nankai District, Tianjin 300193, China; 7 Department of Nephrology, Affiliated Hospital of Liaoning University of TCM, 33 Beiling Street, Huangu District, Shenyang, 110032, China

**Keywords:** Shenyan Kangfu tablets, Efficacy, Safety, Diabetic nephropathy, Randomized controlled trial

## Abstract

**Background:**

Diabetic nephropathy (DN) is a major microvascular complication with diabetes. In China, an estimated 34.7 percent of people diagnosed with diabetes have renal complications and a further 50 percent die of renal failure. Hence, identification of alternative treatments for these patients should be given priority. The Shenyan Kangfu tablet (SYKFT) is a new formulation of an existing and widely acclaimed Chinese herbal tea for treating qi-yin deficiency syndrome. Because a considerable portion of DN patients presenting with symptoms of swelling, fatigue and weak limbs would be diagnosed with qi-yin deficiency syndrome according to the traditional Chinese medicine (TCM) diagnostic criteria, we hypothesize that SYKFT may represent a complementary drug for DN patients with the corresponding syndrome. In view of this, we have designed a trial to assess the efficacy and safety of SYKFT for patients with diabetic nephropathy exhibiting signs of qi and yin deficiency.

**Methods:**

This is a multicenter, double-blind, randomized controlled trial (RCT). The total target sample size is planned at 80 participants, with a balanced (1:1) treatment allocation. The experimental intervention will be SYKFY plus irbesartan (SI regimen) and the control intervention will be a placebo plus irbesartan (PI regimen). Participants will receive two courses of medication treatment each lasting 8 weeks. The primary outcome will be the composite of the quantitative 24-hour urinary protein level and urinary albumin excretion rate (UAER). Changes in urine albumin-to-creatinine ratio (UACR) and DN staging, and TCM symptom improvement will be the secondary outcome measures. Adverse events (AEs) will be monitored throughout the trial.

**Discussion:**

This study will be the first placebo-controlled RCT to assess whether SYKFT plus irbesartan will have beneficial effects on enhancing overall response rate (ORR), changing DN staging, improving clinical symptoms, and reducing the frequency of AEs for DN patients with qi-yin deficiency syndrome. The results of this trial will help to provide evidence-based recommendations for clinicians.

**Trial registration:**

Chinese Clinical Trials Register, Identifier: ChiCTR-TRC-12002182

## Background

Diabetic nephropathy (DN) is renal disease or damage that occurs in patients with diabetes [1]. The World Health Organization defines it as a major microvascular complication with diabetes, and a leading cause of dialysis and kidney transplant in developed countries [2]. The American National Diabetes Fact Sheet 2011 found DN was liable for 44 percent of new cases of renal failure in 2008 in the United States [3]. According to a national survey conducted in 2007 and 2008, China has the world’s largest diabetic population, with more than 92.4 million people affected [4]. The survey also linked 34.7 percent of the Chinese type II diabetic patients with kidney complications [4]. Moreover, the Chinese National Blueprint for Diabetes Prevention and Control 2011 attributed the deaths of half of all diabetes patients complicated with kidney diseases to renal failure [5]. Early intervention is of profound significance to mitigating clinical symptoms, managing proteinuria and hematuria, slowing disease progression and preventing life-threatening kidney failure.

Currently, conventional treatments for DN include lifestyle changes, dietary protein restriction as well as blood pressure and blood glucose control. Antihypertensives and drugs to reduce proteinuria, such as angiotensin-converting enzyme inhibitors (ACEIs) or angiotensin receptor blockers (ARBs), are recommended renoprotective medications for DN sufferers [4]. However, effective as they are, research finds long-term medication use may lead to a number of adverse reactions such as cough, hypotension, hyperkalemia, exacerbated kidney function and also have negative effects on the fetus [6]. As a result, focus has been shifted from western medicine to traditional Chinese medicine (TCM) for an effective integrated approach to DN, with prospectively low expenses and minimum undesired effects.

It is well known that traditional Chinese medicine cures ailments on the basis of the signs and symptoms of a patient [7]. This means the same group of symptoms is likely to be remedied with the same herbal formula, regardless of the disease type (*Bian zheng lun zhi* in Chinese pinyin), a therapeutic rule derived from the wisdom of the Han dynasty herbalist Zhang Zhongjing (A.D. 150 to 219) [7]. Shenyan Kangfu tablets (nephritis recovery tablets, literally, hereafter referred to as SYKFT) is a Chinese patent drug originally designated for clinical treatment of chronic nephritis patients diagnosed with qi-yin deficiency syndrome (a TCM syndrome characterized by the lack of qi and yin energy in the body) [8]. SYKFT has been considered for use in DN treatment because DN patients may share the same TCM syndrome and clinical symptoms with nephritis sufferers. For instance, they may both display symptoms of mental and physical fatigue, swollen face and limbs, sore waist and weak limbs or dizziness and tinnitus, and may both present a red tongue with little coating and a sunken and string-like/thready pulse upon TCM tongue and pulse diagnosis. Moreover, patients suffering from both diseases may develop proteinuria and hematuria with the progression of renal insufficiency. Thus we speculate that the effective solution for the treatment of nephritis may play a similar role in the treatment of DN. We will conduct a multicenter, double-blind randomized clinical trial (RCT) of SYKFT in phase III and IV DN patients to test this hypothesis.

### Objectives

This clinical trial aims to assess whether Shenyan Kangfu tablets combined with irbesartan (SI regimen) is superior to a placebo combined with irbesartan (PI regimen) in efficacy and safety for DN patients with qi-yin deficiency syndrome.

## Method

### Study design

**Figure 1:**
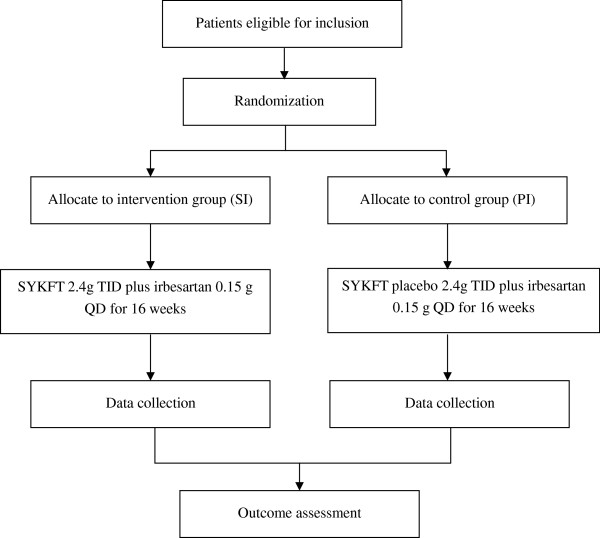
Study flow chart.

This is a multicenter, randomized, double-blind and placebo-controlled clinical trial lasting 16 weeks. The design of the trial integrates strict and scientific clinical research methodology in compliance with principals laid down in the Declaration of Helsinki and the Guidelines for Good Clinical Practice (Figure 1).

### Setting

Participants will be enrolled at five trial sites in four cities in mainland China: 1) Longhua Hospital of Shanghai University of TCM; 2) The Sixth People's Hospital of Shanghai; 3) Guangxing Hospital Affiliated to Zhejiang University of TCM; 4) The First Affiliated Hospital of Tianjin University of TCM and 5) The Affiliated Hospital to Liaoning University of TCM.

### Participants

The study plans to enroll 80 patients diagnosed with diabetic nephropathy and qi-yin deficiency syndrome. They should also meet the items of both the inclusion and exclusion criteria presented in a following section.

### Study criteria

#### Diagnostic criteria

##### Diagnostic criteria for DN

Stage III: early-stage diabetic nephropathy. Urinary albumin excretion rate (UAER) falls within the range of 20 to 200 ug/min or 30 to 300 mg/24h, accompanied by slightly elevated blood pressure.

Stage IV: also termed clinical or dominant diabetic nephropathy. This stage is featured by a large amount of proteinuria, UAER >200 ug/min or the urinary protein quantitative is persistently higher than 500 mg/24h, and it is nonselective proteinuria. Some may present elevated blood pressure and symptoms of nephrotic syndrome.

##### Diagnostic criteria for TCM syndrome differentiation

According to the Guidelines for Clinical Research of Chinese Medicine (New Drug) [9], a patient ought to be currently displaying all the primary symptoms and at least two of the several secondary symptoms listed below to be diagnosed with qi-yin deficiency syndrome.

•Primary signs and symptoms include thirst, increased food and water consumption, excessive hunger and excessive urination at night.

•Secondary symptoms involve exhaustion, fatigue, sore waist, weak legs, swelling face and limbs, dizziness, tinnitus, shortness of breath, tiredness of speaking, sore throat, dry mouth, cold extremities, being upset, insomnia, low-grade fever in the afternoon and feverish palms and soles.

•Tongue and pulse: red tongue with little fur and thready or weak pulse.

### Eligibility and ineligibility criteria

#### Inclusion criteria

•Men and women aged 35 to 65 years.

•Diagnosis of diabetic nephropathy, stage III to IV.

•TCM diagnosis of qi-yin deficiency syndrome.

•Provision of written informed consent by participants or surrogates.

#### Exclusion criteria

•Diagnosis of kidney cancer or other types of renal damage.

•Presence of complications that might affect observation on the curative effects of the test drug.

•Uncontrolled hypertension (blood pressure being persistently greater than 140/90 mmHg).

•Diagnosis of type 1 diabetes mellitus.

•Presence of urinary tract infection.

•Glycated hemoglobin (HbA1c) >59 mmol/mol.

•Twenty-four-hour urinary protein >3 g, serum albumin (ALB) <25 g/L and glomerular filtration rate (GFR) <60 ml/min.

•Presence of serious primary hepatic, pulmonary, hematologic or cardiovascular diseases or any other life-threatening conditions.

•History of allergy to TCM products.

•Recent participation in another clinical trial.

•Pregnancy, lactation or those who are preparing for pregnancy.

### Other criteria

#### Concomitant treatments and forbidden drugs

•Use of any other Chinese medicine or western therapy like ACEIs or ARBs to relieve symptoms of diabetic nephropathy ought to be strictly prohibited.

•Use of medication to control other conditions of the patients, such as anticoagulatory or lipid-lowering drugs, is allowed.

•Details of any additional drug or therapy taken must be recorded in the case report form (CRF). These include drug name, dose and treatment duration.

Early suspension or termination of the trial will occur in the following situations:

•Occurrence of serious adverse events, flawed protocol or significant deviation in implementation.

•Funding or management problems.

•The administrative authorities decide to terminate the study.

Patients will be withdrawn or be rejected for further participation on the grounds below:

•Voluntarily quitting.

•Use of forbidden medication or treatment during the trial that might affect analysis of results.

•Early termination of the process based on the investigator’s judgment (in the case of misdiagnosis, development of severe complications, and so on.).

•Failure to keep the patient blinded, or deblinding in case of emergency.

### Exit criteria corresponding to treatment failure

•Uncontrolled blood pressure.

•HbA1C levels >59 mmol/mol.

•Uncontrolled blood glucose.

•Persistently elevated proteinuria.

### Interventions

Participants randomized to the treatment group will be administered SYKFT 2.4 g three times daily plus irbesartan 0.15 g once daily for 16 weeks. Those in the control group will receive SYKFT placebo 2.4 g three times daily in addition to irbesartan 0.15 g once daily for 16 weeks. Both groups will receive behavioral intervention and health education such as diet instructions. All test drugs, that is SYKFT, the placebo and irbesartan (trade name: Anbowei in Chinese pinyin) are manufactured by Tianjin Tongrentang Pharmaceuticals.

### Measurement items and time points of data collection

Relevant examinations will be performed and patient data will be collected at 2 weeks before baseline, at baseline, at 2 weeks post baseline and every 4 weeks from 4 to 16 weeks during treatment. Items to be measured and the time points of data collection can be found in Table 1.

**Table 1:** Measurement items and point of data capture

	**Screening**	**Baseline**	**Treatment**				
	**Visit 1**	**Visit 2**	**Visit 3**	**Visit 4**	**Visit 5**	**Visit 6**	**Visit 7**
**Items**	**−2 weeks**	**0 weeks**	**2 weeks**	**4 weeks**	**8 weeks**	**12 weeks**	**16 weeks**
Informed consent form	X						
Inclusion/exclusion criteria	X						
Demographic information	X						
Medical/drug use history	X						
Concomitant disease and treatment	X						
General physical examination	X						
Combined medication	X	X	X	X	X	X	X
Vital signs	X	X	X	X	X	X	X
TCM symptom	X	X	X	X	X	X	X
Urine protein test	X	X	X	X	X	X	X
Routine urine test	X	X	X	X	X	X	X
Renal function (SCR, BUN, GFR) Serum albumin	X	X	X				X
Routine blood test	X	X					X
Stool routine test and fecal occult blood test	X	X					X
Liver function (ALT/AST/GGT/TBIL/ALB)	X	X					X
Blood electrolytes	X	X	X				X
Blood coagulation (TT/APTT/PT/FIB)	X	X					X
Blood glucose	X	X	X	X	X	X	X
Glycosylated hemoglobin	X	X			X		
Electrocardiogram (EGG)	X	X					X
Adverse events	X	X	X	X	X	X	X
Randomization		X					
Drug distribution	X	X	X	X	X	X	
Drug recycling		X	X	X	X	X	X
Drug count		X	X	X	X	X	X
Research conclusion							X

X: Have indicators tested in the specific time period. ALB, serum albumin; ALT, alanine aminotransferase; APTT, activated partial thromboplastin time; AST, aspartate aminotransferase; BUN, blood urea nitrogen; FIB, fibrinogen; GFR, glomerular filtration rate; GGT, gamma-glutamyl transpeptidase; PT, prothrombin time; SCR, serum creatinine; TBIL, total bilirubin; TCM, traditional Chinese medicine; TT, thrombin time.

### Outcome measurements

#### Primary outcomes

The primary outcome of this trial will be the composite of the quantitative 24-hour urinary protein levels and urinary albumin excretion rate (UAER).

#### Secondary outcomes

Secondary outcomes include changes in urine albumin-to-creatinine ratio (UACR), TCM symptom improvement and changes in diabetic nephropathy staging (for example, from phase III to IV).

#### Safety outcomes

Adverse events will be monitored throughout the trial and several biological indicators (routine blood and urine test, stool routine test, electrocardiogram (ECG), liver function and blood coagulation) will also be closely watched.

### Measurement tools

#### Measurement scale for TCM symptoms

The measurement scale for TCM symptoms recommended by the Guidelines for Clinical Research of Chinese Medicine (New Drug) [9] will be used for ease of assessment. Each of the primary symptoms necessary for the diagnosis of qi-yin deficiency syndrome will be scored 0, 2, 4 or 6, while a secondary symptom is scored 0, 1, 2 or 3, and the scores then summed to yield a total score of both types of symptoms for a patient. It is stipulated that the total primary symptom score shall not exceed 18, and the total score for secondary symptoms shall not exceed 33 for an individual patient.

### Efficacy assessment tools

#### For TCM symptoms

Following the Guidelines for Clinical Research of Chinese Medicine (New Drug) [9], the reduction in the total TCM symptom score of the patient will be calculated and used as an efficacy indicator (EI) for the evaluation of treatment efficacy. EIs will be calculated according to the following formula:

$$\begin{array}{l} \mathrm{EI}=\left(\mathrm{Total}\;\mathrm{symptom}\;\mathrm{score}\;\mathrm{at}\;\mathrm{baseline}\right)\\ \left(\;-\mathrm{Total}\;\mathrm{symptom}\;\mathrm{score}\;\mathrm{post}\;\mathrm{treatment}\right)/\\ \;\mathrm{Total}\;\mathrm{symptom}\;\mathrm{score}\;\mathrm{at}\;\mathrm{baseline}\times 100\% \end{array}$$

The degree of symptom improvement will be presented in four categories ranging from ‘full recovery’ (EI ≥90%), ‘good recovery’ (90% >EI ≥70%), ‘modest recovery’ (70% >EI ≥30%) to ‘no recovery’ (EI <30%).

### For laboratory indicators of the disease

Full recovery (FR): the quantitative 24-hour urinary protein, UAER and renal function return to normal levels.

Good recovery (GR): a decrease of ≥40% in UAER or the quantitative 24-hour urinary protein.

Modest recovery (MR): a decrease of <40% in UAER or the quantitative 24-hour urinary protein.

No recovery (NR): unchanged or worsened laboratory test results.

To assess efficacy with regard to either TCM symptom improvement or amelioration of clinical signs, an overall recovery rate (ORR) will be calculated using the following formula.

$$\begin{array}{l} \mathrm{ORR}=\left(\mathrm{FR}\;\mathrm{count}+\mathrm{GR}\;\mathrm{count}+\mathrm{MR}\;\mathrm{count}\right)/\\ \;\mathrm{Group}\;\mathrm{population}\times 100\%. \end{array}$$

### Reporting of adverse events

Adverse events (AEs) will be recorded in medical diagnostic terminology. Detailed symptoms, time of occurrence, duration, severity, possible causal relationships, actions taken, results and other relevant information will be reported. In the case of an adverse event, researchers must fill out a serious adverse event (SAE) form and notify both the institutional review board (IRB) and the regulatory authorities within 24 hours.

### Sample size calculation

This is an exploratory phase II clinical study designed to provide evidence and a scientific rationale for advancing the treatment regimen into a phase III confirmatory study. The planned sample size is 80, with 40 in each treatment group.

### Randomization and allocation concealment

A total of 80 patients will be randomly assigned in a ratio of 1:1 to either the SI or the PI group using a computer-generated random number sequence. To guarantee rigorous methodology, center-stratified randomization and central coordination will be initiated. The central randomization system will assign a unique code to each newly included participant, who will then be administered the corresponding treatment regimen.

An expert statistician of the Tianjin Institute of Clinical Evaluation and a staff member from the sponsoring organization will act as the coder, and they will be shielded from patient recruitment. The trial management board will be responsible for keeping the blinding codes and other information confidential.

### Blinding

This trial will use the double-blind design. The placebo tablets are indistinguishable from SYKFT in shape, size, color and packaging. Thus, both participants and clinicians will be masked. Moreover, a designated pharmacist not involved with the study will be held responsible for handing out medication.

### Statistical analysis

The statistical analysis will be performed by a statistician with the Tianjin Institute of Clinical Evaluation, and the data will be analyzed using Statistical Analysis System (SAS) software version 9.2 (SAS Institute Inc., Cary, NC, USA). A formal and detailed statistical analysis plan will be documented prior to data locking.

### Efficacy assessment

All primary analyses for this study will strictly follow the intention-to-treat (ITT) principle. The full analysis set (FAS) population, which will include randomized subjects who have received treatment at least once, will be used for primary efficacy outcomes analysis. In addition, a per-protocol analysis will be performed on the secondary outcomes. The per-protocol set (PPS) population will be restricted to patients who are compliant with the treatment protocol and have a completed CRF. Good compliance indicates the drug actually taken equals 80 to 120 percent of the required dosage. Missing values will be replaced using the last observation carried forward (LOCF) method.

Continuous variables will be described as mean ± standard deviation, and dichotomous data will be reported as percentages. A two-tailed test will be applied. Statistical significance will be defined as *P* <0.05 and 95% confidence interval will be calculated. Heterogeneity between groups will be tested at baseline.

The main analysis will be a comparison between the two groups concerning the proportion of patients achieving different levels of recovery (FR, GR, MR or NR) in terms of TCM symptoms and clinical biomarkers using the Wilcoxon rank sum test. In addition, the ORR will be compared between the two arms using the center-stratified Cochran-Mantel-Haenszel (CMH) method. For secondary outcomes, all continuous data will be analyzed via analysis of covariance adjusted for clinical center and baseline. Analyses of binary data will be based on logistic regression models.

### Safety assessment

The safety set (SS) population, which will include randomized subjects who have made at least one visit, will be used for safety analysis. The frequency of adverse events and incidence of abnormalities in laboratory tests before and after treatment will be compared for the two groups using a chi-squared test. Any possible causal relationship between the test drug and the abnormality will be detected using an appropriate statistical model

### Management of test drugs

#### Drug packaging

To ensure effective blinding, SYKFT and the placebo will be prepared respectively but uniformly packaged. On the outside of the package, the drug code, name, specifications, function and indications, usage and dosage, storage conditions and name of the manufacturer will be specifically marked and a tag indicating ‘for trial use only’ will be attached.

### Storing, handing out and reclaiming of test drug

A drug administrator will be assigned at each center, who will be responsible for the storage, handing out and reclaiming of the test drug. They shall make sure the test drug is kept in a dry, ventilated place and at the appropriate temperature. The test drug will be dispensed during the treatment period and reclaimed at study completion. The drug administrators should return unused drugs to the sponsor or destroy them as required. Upon drug destruction, they should make records and file a ‘Certification of Test Drug Destruction’.

### Quality control of data

Quality control of the research data will be achieved by rigorous monitoring throughout the trial process. Trial monitors will pay regular visits to each site, where they will recheck CRFs, inspect the storage of the investigational drugs and review investigators’ records. After verification of the content of the written CRFs, data will be collected and inputted into a database by two full-time research staff independently. The standard operating procedures (SOP) will be invariably followed.

### Ethics issue

This study has been approved by the IRBs of each participating hospital, and is registered at ChiCTR (http://www.chictr.org, trial identifier ChiCTR-TRC-12002182). It will be made sure only clinicians holding the necessary qualifications act as principal investigators. Written informed consent will be obtained from individual participants prior to enrollment.

## Discussion

SYKFT is an improved version of the herbal prescription based on the valuable therapeutic knowledge of the late TCM practitioner Zhao Enjian, who had enjoyed nationwide fame for treating patients with chronic nephritis [10]. The formula is a combination of 11 herbal ingredients with a synergic effect of tonifying qi and yin, nourishing the kidney and spleen and detoxicating the body [11]. The tablet formulation is manufactured by Tianjin Tongrentang Pharmaceuticals (Register No: Z10940034) and film-coated tablets are now available for diabetes patients [10]. SYKFT is recommended for treating a variety of indications including fatigue, waist pain, swollen face and limbs, dizziness, tinnitus, proteinuria and hematuria.

Preclinical study evidences that SYKFT has curative effects on streptozotocin-induced diabetic rats with unilateral nephrectomy [12]. Another research study finds the drug’s renoprotective effect is associated with the upregulation of podocalyxin expression in DN mouse models [13]. The chronic toxicity test with an observation period of six months reported no death and no abnormal pathological changes in the experimental animals [unpublished data, Tianjin Institute of Medical and Pharmaceutical Science]. Also, SYKFT has been reported to have improved symptoms, controlled proteinuria and retarded the progression of renal failure for DN patients in clinical observations and preliminary trials [14-22].

In view of the current evidence base, we have designed this placebo-controlled RCT to test the efficacy and safety of SYKFT for diabetic nephropathy patients with qi-yin deficiency syndrome. The findings of this study will inform hypothesized effects of SYKFT and may provide an alternative treatment option for diabetic nephropathy. It will also provide evidence and a scientific rationale for advancing the treatment regimen into a phase III confirmatory study.

## Trial status

The trial was initiated in November 2012 and is currently open for enrollment. A total of 20 participants had been enrolled and randomized for this trial by March 2013.

## Abbreviations

ACEIs: Angiotensin-converting enzyme inhibitors; AEs: Adverse events; ALB: Serum albumin; ALT: Alanine aminotransferase; APTT: Activated partial thromboplastin time; ARBs: Angiotensin receptor blockers; AST: Aspartate aminotransferase; BUN: Blood urea nitrogen; CRF: Case report form; DN: Diabetic nephropathy; ECG: Electrocardiogram; EI: Efficacy indicator; FAS: Full analysis set; FIB: Fibrinogen; FR: Full recovery; GFR: Glomerular filtration rate; GGT: Gamma-glutamyl transpeptidase; GR: Good recovery; IRB: Institutional review board; ITT: Intention-to-treat; LOCF: Last observation carried forward; MR: Modest recovery; NR: No recovery; ORR: Overall recovery rate; PI: Placebo and irbesartan; PPS: Per-protocol set; PT: Prothrombin time; RCT: Randomized controlled trial; SAE: Serious adverse event; SCR: Serum creatinine; SI: SYKFT and irbesartan; SOP: Standard operating procedure; SS: Safety set; SYKFT: Shenyan Kangfu tablet; TBIL: Total bilirubin; TCM: Traditional Chinese medicine; TT: Thrombin time; UACR: Urinary albumin-to-creatinine ratio; UAER: Urinary albumin excretion rate

## Competing interests

The authors declare they have no competing interests.

## Authors’ contributions

All authors have made substantive intellectual contribution to this study in regard to conception and design, or acquisition of data, or analysis and interpretation of data. HCS conceived of the study and revised the manuscript critically for important intellectual content. HW and WM drafted the manuscript and made revisions. HW, SJM and JW participated in the design of the study and will be responsible for the trial management. WM and DMX have been involved in the coordination of the trial. JBZ helped to draft the manuscript and will be responsible for statistical data analysis. YYD, NSW, HYC, HTY and XHH have been involved in patient recruitment and treatment. All authors have read and approved the final manuscript.

## References

[B1] The Health Central website [http://www.healthcentral.com/ency/408/000494.html]

[B2] The World Health Organization website [http://www.who.int/diabetes/action_online/basics/en/index3.html]

[B3] The American Diabetes Association website [http://www.diabetes.org/diabetes-basics/diabetes-statistics/]

[B4] The Chinese Diabetes Society of the Chinese Medical Association The Chinese national guideline for type II diabetes prevention and treatment 2011 Chin J Endocrinol Metabol 2011 27 Suppl 12 45

[B5] Advisory Committee on Diabetes Prevention and Control The Chinese national blueprint for diabetes prevention and control Tongji Med Coll of Huazhong Univ Sci Technol 2011 11 14

[B6] The K/DOQI Clinical Practice Guidelines on Hypertension and Antihypertensive Agents in Chronic Kidney Disease [http://www.kidney.org/professionals/kdoqi/guidelines_bp/guide_11.htm] 15114537

[B7] Zhang ZJ Treatises on Cold Injury 2005 Beijing: People’s Medical Publishing House

[B8] Xie XS Fan JM Li HJ Deng Y Zhong X Evaluating the use of Shenyan Kangfu tablets in treating kidney diseases Chin J Integr Tradit West Nephrol 2007 8 493 494

[B9] Zheng YY Guidelines for Clinical Research of Chinese Medicine (New Drug) 2002 Beijing: Chinese Medicine and Science Publication House

[B10] Liu JJ Zhou LH Li YC Bai MF Research on the film-coating techniques for Shenyan Kangfu tablets Chin Herb Med 2000 31 591 592

[B11] The TCM Shop Online website [http://www.tcmshoponline.com/shen-yan-kang-fu-pianforchronic-nephritis-deficiencyqiyin-p-329.html]

[B12] Wang L Miao SJ Li X Chong YY Wang J Chen WP Pharmacodynamic effects of Shenyan Kangfu tablets on experimental diabetic nephropathy in rats Chin Trad Patent Med 2012 11 29 32

[B13] Wei HK Fang JG Sun YY Zhang XD Effect of Shenyan Kangfu tablet on podocalyxin in renal tissue of rats with diabetic nephropathy Chin J Integr Tradit West Nephropathy 2009 10 485 488

[B14] Xie FJ Zhao J Wang SY Clinical observation on the effects of Shenyan Kangfu tablets for diabetic nephropathy treatment Chin J Gen Pract 2012 10 123 124

[B15] Xin SQ Clinical observation of Shenyan Kangfu tablets for diabetic nephropathy in 22 cases Chin J Integr Tradit West Nephropathy 2010 11 450 451

[B16] Shu F Du MX Zhang BG Shenyan Kangfu tablets for elderly type II diabetic nephropathy patients Practical Clin J Integrated Tradit Chin West Med 2010 10 24 25

[B17] Xu J Chen WL Clinical observation of Shenyan Kangfu tablets for diabetic nephropathy Mod J Integr Tradit Chin West Med 2009 18 374 375

[B18] Guo W Dan G Chen Y Xue P Lu R Comparative study of therapeutic effects of Shenyan Kangfu tablet and candesartan cilexetil on diabetic nephropathy Med J Natl Defending Forces in Southwest China 2009 19 681 683

[B19] Du MX Shu F Zhang BG Clinical observation on Shenyan Kangfu tablets for type II diabetic nephropathy Chin J Integr Tradit West Nephropathy 2007 8 606 607

[B20] Shu WH Chen HZ Zhang JF Clinical study on Shenyan Kangfu tablets for diabetic nephropathy Guide Chin Med 2008 6 46

[B21] Deng YY Chen YP Tang H Xu RJ Jin YM Zhu R Guo Q Ge FF Clinical observation on Shenyan Kangfu tablets for diabetic nephropathy Chin J Integr Tradit West Nephropathy 2005 6 151 153

[B22] Li W Observation on the effects of Shenyan Kangfu tablets for diabetic nephropathy prevention and treatment Chin J Prev Control of Chronic Non-Communicable Dis 1998 6 201

